# The evolution and stability of multi-ethnic residential neighbourhoods in England

**DOI:** 10.1111/tran.12416

**Published:** 2020-09-29

**Authors:** Gemma Catney, Richard Wright, Mark Ellis

**Affiliations:** 1 Geography, School of Natural and Built Environment, Queen’s University Belfast, Belfast, UK; 2 Department of Geography, Dartmouth College, Hanover, NH, USA; 3 Department of Geography and Center for Studies in Demography Ecology, University of Washington, Seattle, WA, USA

**Keywords:** diversity, England, ethnicity, mixing, neighbourhood, race

## Abstract

This paper analyses the most ethnically diverse spaces in England. We define multi-ethnic neighbourhoods as spaces where no one group is in a majority and at least five ethnic groups have representation. Around 4% of all English neighbourhoods (Lower Layer Super Output Areas) met these criteria in 2011. Often mislabelled as “segregated” spaces, the growth of ethnically diverse neighbourhoods helps benchmark increased inter-ethnic contact, yet we know very little about their spatial extent and the dynamics of their expansion. We use Census data for 1991, 2001, and 2011 to consider how neighbourhood-level diversity has changed during a period of substantial increase in ethnic diversity at the national scale. To what extent did these highly diverse areas grow, and what is the geography of that growth? Which types of areas did these neighbourhoods transition from? For example, were multi-ethnic neighbourhoods formerly low or moderately diverse, and which groups dominated these locales? We also consider if multi-ethnic neighbourhoods are here to stay, or if they are compositionally unstable. We reveal a surprising aspect in England’s neighbourhood transitions: multi-ethnic neighbourhoods are highly stable, and increasingly so. Some 88% of neighbourhoods that were multi-ethnic in 1991 retained their high-diversity status in 2001, while over 95% of 2001 multi-ethnic neighbourhoods remained highly diverse by 2011. This is a different story to that of the USA, where high-diversity neighbourhoods have received more scholarly attention, and where these neighbourhoods have high attrition rates, functioning as stepping stones to another type of space. We explore the demographic and housing dynamics associated with this stability.

## 1 ∣ INTRODUCTION

Multi-ethnic neighbourhoods are a new and growing feature of urban landscapes across the Global North (e.g., Harris, 2018; Johnston et al., 2013; Pratsinakis et al., 2017; Wright et al., 2020a). They are important places to study not only because they are novel, but also because they offer heightened possibilities for understanding inter-ethnic contact, mixing, and interaction. In the UK, research on these neighbourhoods has usually been through case studies, which have provided rich insights into attitudes to “commonplace” diversity and “everyday” encounter (Wessendorf, 2013), the relationships between diversity, place belonging, and identity (Frost & Catney, 2020), the new layers of diversity in urban locales (Platts-Fowler & Robinson, 2015), and migrant acceptance and celebration of diversity (Hickman et al., 2008).

We have a complementary interest. This paper reports on the results of a large-scale quantitative analysis of the dynamics of multi-ethnic neighbourhoods in England, what we take to be highly diverse places in which at least five ethnic groups have meaningful representation. We paint a picture of England’s ethnic landscape and explore how this has altered between 1991 and 2011 – a period of major population, policy, and political shifts. Against this backdrop, we ask: To what extent did the number of multi-ethnic residential neighbourhoods increase, and what is the geography of that growth? Who is exposed to high diversity, and how has that changed over time? Are multi-ethnic neighbourhoods transitory spaces, and what demographic dynamics are associated with their (in)stability?

Several factors spur our curiosity about the transitions associated with multi-ethnic neighbourhoods. High diversity cannot be understood as a fixed state – it must emerge, and it may also dissipate. Social science research on neighbourhood dynamics has a long history. Most recent studies have moved from binary thinking about Black–White transitions to accounting for multiple groups in contexts of both segregation and diversity (e.g., Logan & Zhang, 2010). Despite critiques, the UK literature continues to lean on US-based models for inspiration. In the USA, highly diverse neighbourhoods have been found to be spaces of transition, with one study finding that less than 50% of high-diversity neighbourhoods remained so for more than a decade (Wright et al., 2020a). We might expect differences across the Atlantic given, for example, variations between the USA and UK in immigration, histories of racial subordination, and geographic scale. Yet we might also anticipate that high-diversity spaces evolve in similar ways. If high neighbourhood diversity in England is stable, rather than transitionary, this would disrupt narratives that these multi-ethnic places are paths to polarisation (e.g., Casey, 2016; cf. Logan & Zhang, 2010).

The paper begins with a brief review of the literature concerning neighbourhood transition theory and recent changes in the ethnic and racial make-up of UK neighbourhoods. We then explain our neighbourhood classification scheme, and show its application to neighbourhood transitions in England between 1991 and 2011. English neighbourhoods remain predominantly White, but we show how this is changing and that one of the main pathways is towards multi-ethnicity. Accordingly, we devote much of the analysis to offering insights into why multi-ethnic spaces came about, which groups are in them, and their stability. The conclusions broaden the discussion to contemplate the implications of our findings for inter-ethnic mixing.

## 2 ∣ NEIGHBOURHOOD TRANSITIONS AND ETHNIC MIXING

Many approaches to neighbourhood (ethnic) transition trace to the USA and the mass-migration period in the early 20th century. Just as they do today, the majority of newly arrived immigrants settled in major metropolitan areas. One of these, Chicago, became the place where influential scholars studied the profound changes at work in urban space (e.g., Park et al., 1925). Among their interests lay a concern with the neighbourhood transitions of both people (socio-spatial mobility) and place (rapidly changing urban landscapes). Park and colleagues argued that the in-migration and subsequent growth of a new group corresponded with the out-migration of a previously settled group to new, generally more desirable (e.g., suburban), neighbourhoods. Original immigrant enclaves were altered again by subsequent waves of newcomers.

The accent at the time was on neighbourhood turnover, not on ethnic and racial mix. Massey and Denton (1993) reassessed this moment, and Chicago School Theory, in two different ways. They pointed out that immigrant enclaves, the “Little Italys” and the “Magyar” district in early 20th-century Chicago, actually contained many different national-origin groups (1993, p. 32). At the same time, the growing numbers of Black people arriving in Chicago from the US South became concentrated in segregated spaces, reflecting their distinctive racialised subordination. Into the mid-century, European immigrants and their descendants melded into an enlarged White category, ushering in a new era of research on neighbourhood dynamics that foregrounded the binary of Black–White segregation. Neighbourhood mix became associated with tipping points, the moment at which the Black share of a neighbourhood’s population triggered White flight (Schelling, 1971). Much recent neighbourhood transition research, as well as scholarship on ethnically differentiated socio-spatial mobilities, frame their theoretical contributions with at least a nod to Chicago School-derived spatial assimilation (and allied) theories, including studies in European contexts (e.g., Coulter & Clark, 2019; McAvay & Safi, 2018; Vogiazides & Chihaya, 2020; Wessel et al., 2017; Zuccotti, 2019).

Increased immigration in the last few decades has produced demographic change across the Global North. In the USA, as most new immigrants are Latinx or Asian, different racial and ethnic spatial dynamics are in play, requiring a reassessment of earlier methods and theory (e.g., Logan & Zhang, 2010). Overall residential segregation levels are down, and mixing in neighbourhoods, especially those that were over 80% White, is on the rise (Ellis et al., 2018). At the same time, many newly arrived immigrants and their offspring residentially cluster, many Black people continue to live in relative isolation, and avoidance of Black residential spaces by other people remains commonplace. And while the number of highly diverse, multi-ethnic neighbourhoods has grown, they remain the exception, concentrated in relatively few metropolitan areas and likely not to last. The attrition rates are considerable. In the USA, these neighbourhoods largely transition to moderately diverse Latino-dominated neighbourhoods, but also to moderately diverse White tracts – hypothesised as an outcome of gentrification (Wright et al., 2020a).

The UK has a different history of immigration, demographic change, colonialism, and racial and ethnic relations. Nevertheless, the foreign-born proportion of the UK population was, in 2019, similar to that of the USA (~14%). Also, as in the USA, Black, Asian and Minority Ethnic (BAME) populations have increased their overall share, and there has been a discernible reduction in levels of residential segregation (Catney, 2016a; Harris, 2014; Johnston et al., 2013). So how might these similarities and differences translate across national contexts, in terms of neighbourhood transition and mixing?

Johnston et al. (2013) were among the first to examine the extent of, and changes in, neighbourhood ethnic mixing in England and Wales. Exploring Census data for Output Areas between 2001 and 2011, they distinguished five neighbourhood types based on the balances of their White and non-White populations. According to their schema (Johnston et al., 2007), mixing is identified when the White majority dominate at either 50%–80% of the neighbourhood but BAME groups form a substantial minority (their “Type 2” neighbourhood), or BAME groups dominate at 50%–70% of the population but members of the White group form a substantial minority (“Type 3” neighbourhood). Using this classification of area types, they illustrated the “increased multi-ethnic character of many residential neighbourhoods, especially in London” (2013, p. 758). The share of people in their Type 3 neighbourhood, with high ethnic diversity most akin to that measured in this paper (see section 3), nearly doubled between 2001 and 2011 in England and Wales, and, in London, grew the fastest of all neighbourhood types.

Johnston et al. (2015) expanded on their (2013) analysis, underscoring two points pertinent to this paper: (1) places where White people were numerically dominant in 2001 became more mixed in 2011, and (2) in those places where White people were in a minority in 2001, their numbers dwindled a decade later, “but without the development of separate enclaves where one Non-White group predominates” (2015, p. 38). That is, they noted the ascendance of their Type 3 neighbourhood over other neighbourhood types, including neighbourhood types where one or more BAME group formed a majority.

Shifting scale to local authorities, Simpson (2015) analysed “plural cities,” defined as when no ethnic group (White or minority) equals or exceeds 50%. Exploring 2011 Census data for England, Wales and Scotland, Simpson categorised 23 (of 33) of London’s boroughs as plural, as well as London as a whole, its nearby local authorities of Slough and Luton, and Leicester. The analysis demonstrated how “Britain’s cities are more ethnically diverse than ever before … cities labelled by politicians as ‘segregated’ are, in fact, the most diverse” (Simpson, 2015, p. 49). Catney (2016b) adopted a more fine-grained spatial lens through which to explore ethnic diversity. Using entropy to measure diversity levels in Census wards in England and Wales for 1991–2011, she showed that it is not only cities that have experienced a growth in ethnic diversity. Expanding Rees and Butt’s (2004) study of ethnic diversity in England between 1981 and 2001, Catney detected a relatively modest yet notable deconcentration of ethnic minority populations to suburban and rural locales not traditionally associated with ethnic mixing.

London is demonstrably special in this context. London’s “diversity of diversity,” including and beyond ethnic affiliations (to immigrant origins, languages spoken, religions practised, and so on), means that it is often described as “the world in one city” (GLA, 2018; Vertovec, 2007). The capital is home to by far the greatest concentration of ethnic diversity compared to anywhere else, and, as already noted, this ethnic diversity had steadily increased over time (Catney, 2016b; Johnston et al., 2013).

None of the aforementioned studies focus on highly diverse, multi-ethnic neighbourhoods. Accordingly, we connect the growing interest in high-diversity neighbourhoods in the USA (e.g., Farrell & Lee, 2011; Friedman, 2008; Logan & Zhang, 2010; Wright et al., 2020a, 2020b; Zhang & Logan, 2016) to multi-ethnic neighbourhoods in a UK context. Is high diversity a stepping stone to other neighbourhood types, as is the case in the USA?

## 3 ∣ METHODS AND DATA

The literature concerned with the measurement of multi-ethnic spaces is almost as diverse as the mix it seeks to capture (e.g., Stillwell & van Ham, 2010; Wright et al., 2020b). To understand the geographies and characteristics of multi-ethnic neighbourhoods in England, we adapt a neighbourhood classification scheme that, more broadly, categorises all neighbourhoods according to their levels of diversity and group dominance. While several area classification schemes are available, it is essential that we capture high diversity. Therefore, we follow Holloway et al. (2012), which offers several benefits over other schemas.

Holloway et al. (2012) developed the taxonomy to characterise the changing dynamics of racial segregation and racial diversity at the census tract scale in the USA. They showed that many metropolitan areas were home to neighbourhoods that were becoming increasingly diverse, as well as others that remained stubbornly racially segregated. A neighbourhood might alter its category in several ways; for example, it could change from one level of diversity to another (for example, low- to moderate-diversity) from group differentials in net-migration, birth, and death rates, or the dominant group might instead (or in addition) change. As the schema provides an absolute, rather than relative, measure of diversity, small areas can be compared, providing insight into ethnic geographies across the whole country, and over time. This research team has applied the technique nationally, at state level, across large metropolitan areas and in neighbourhoods (Wright et al., 2014), exploring the shifting nature of White space (Ellis et al., 2018a; Ellis, et al., 2018b) and the instability of highly diverse neighbourhoods (Wright et al., 2020a).

To adapt this taxonomy for the English context, we must account for the differences in census classifications of the English and US cases. To do this, we adjust the six-group entropy-based schema of Holloway et al. (2012) for one based on eight categories. Neighbourhood entropy is defined as:

(1) $$E_{j}=s\left(-\sum_{i=1}^{k}(p_{i}\times ln(p_{i}))\right)$$

where *E*_*j*_ is the entropy of neighbourhood *j* and *p*_*i*_ is the proportion of group *i* in a given area, and there are *k* groups in the area (*k* = 8 in our case). A scaling constant *s* equal to 1/*ln*(*k*) is included so that *E*_*j*_ ranges from 0 to 1. Like Holloway et al. (2012), we identify three types of diversity: low, moderate, and high. After experimentation, *low-diversity* neighbourhoods have a scaled entropy value of less than or equal to 0.3436, or when one ethnic group makes up equal to or greater than 80% of the population of that neighbourhood. *High-diversity*,xs or multi-ethnic, neighbourhoods have a scaled entropy of equal to or higher than 0.6766, and no group constitutes higher than 45% of the neighbourhood’s population. These criteria ensure people from *at least five* groups must be present in these neighbourhoods. They also require that ethnic groups other than the largest two constitute, in combination, at least 20% of that neighbourhood’ s population, with this minimum percentage inversely dependent on the number of groups (e.g., 20.0% if all of our eight groups are present, increasing to 31.2% when only five groups are present). The remaining neighbourhoods are classified as *moderate-diversity*. For low- and moderate-diversity neighbourhoods, we also identify which ethnic group is numerically dominant. Based on the neighbourhood’s largest group, often with well over a majority share of the population, the resultant classification labels include, as examples, “moderate-diversity Indian” and “low-diversity White.” This step makes less sense for neighbourhoods classified as highly diverse (multi-ethnic), since no group can exceed 45% of a neighbourhood’ s population for the area to qualify for this category.

This schema has several features that make it particularly apt for our study. It imposes a demanding definition of high diversity. Some other studies of multi-ethnic neighbourhoods, for example, include spaces that can be majority White (e.g., Walton & Hardebeck, 2016). Unlike some other approaches, the schema also does not pivot around the presence/absence of White people, nor does it presume that the absence of White people is a sign of diversity; neighbourhoods can be majority minority, but still dominated by a single (BAME) group. Despite evidence to the contrary, a narrative persists that (mis) understands ethnic *diversity* as *segregation*, interpreting diverse places that lack a White majority as evidence of separation (compare Kaufmann & Cantle, 2016 with Catney, 2016c, and Khan, 2016). But multi-ethnicity need not, nor should not, centre on the relative presence or absence of White people. For us, multi-ethnic neighbourhoods may include the significant presence of White people, or not. We take the “multi” in multi-ethnic seriously by requiring at least five different groups in a multi-ethnic neighbourhood and substantial representation by the smaller groups.

We recognise that the ethnic group categories we use obscure their internal diversities: ethnic groupings such “Indian,” “Black African,” and “Pakistani” each encompass large variation in culture. The same applies to “White,” by far the largest ethnic group we include. With recent immigration, “Whiteness” in the UK has arguably never been more complex (e.g., Walsh, 2019a, 2019b) or politicised (e.g., Favell, 2020; and see the *Ethnicities* review symposium debating Kaufmann’s 2018 *Whiteshift*^1^). Consequently, we spend some time unpacking this particular grouping in our analysis.

Ethnic group categories are inconsistent between the 1991, 2001, and 2011 Censuses. Among other alterations, the broad White ethnic category was disaggregated into three sub-groups in 2001, and four in 2011. Since 2001, given an increasingly mixed population, four mixed ethnicity categories were included. These changes yielded the categorisation of 16 ethnic groups in 2001, and 18 in 2011, increasing from 10 when the ethnic group question was first introduced in the 1991 Census. Ethnic groups must therefore be aggregated for analyses that cut across time periods. Simpson et al. (2016) made recommendations on the ethnic groups that are most comparable, and we follow their guidance here, thus including in our analyses the ethnic groups White, Indian, Pakistani, Bangladeshi, Chinese, Black African, and Black Caribbean. A residual aggregate “Other” category, not strictly comparable over time (given inconsistencies between the three Censuses in, for example, ethnic group categorisation and self-identification; Simpson et al., 2016), constitutes the eighth. This category’s heterogeneity, and related size, will of course mask some of the finer-grained detail of neighbourhood diversity, but this is unavoidable given temporal changes in ethnic group categorisation. Taken together, these eight ethnic groups sum to the total population. Our study shines a spotlight exclusively on England since, given its considerably greater ethnic diversity than Wales, Scotland, and Northern Ireland, it contains nearly all of the UK’s multi-ethnic neighbourhoods.

Neighbourhoods are defined using the Census statistical zones of Lower Layer Super Output Areas (LSOAs). Introduced in 2001, these units were designed so that each zone had approximately the same size of population. This provides an advantage over Census wards, which are much more variable. In 2011, England had 32,844 LSOAs, with a mean size of 1,614 people. They are the best unit for this study because they offer an appropriate balance of spatial granularity compared to smaller (Output Area) or larger (Middle Layer Super Output Area) zones. The former, with a mean population of 300 people, would be too small to allow us to identify any meaningful diversity, while the latter zones are too large to be considered a “neighbourhood.” We harmonised the ethnic group information into 2011 boundaries for all three census years.^2^ Data processing and analyses were undertaken using the R software environment (R Core Team, 2018).

## 4 ∣ NEIGHBOURHOOD TYPES AND THEIR GEOGRAPHIES

Table 1 shows the number and proportion of England’s LSOAs in each neighbourhood category in 1991, 2001, and 2011. The persistent dominance of White spaces is immediately apparent. In 1991, over 90% of neighbourhoods were low-diversity, majority White. In 2011, this neighbourhood type was still by far the most common, but its share had dropped to 78%. In contrast, neighbourhoods that were also White majority but moderately diverse increased their share, rising from around 8% of England’s neighbourhoods in 1991 to 16% in 2011. Fusing low- and moderate-diversity White neighbourhoods together, just 6% of 2011 neighbourhoods were not White-majority. Despite this, the ethnic diversification experienced by England at the “national” level (Simpson & Jivraj, 2015) is reflected at the neighbourhood scale. The emergence of new neighbourhood types testifies to this diversification. While in 1991 there were no low-diversity Pakistani, low-diversity Bangladeshi, or moderate-diversity Black African neighbourhoods, they could be found in 2001 and 2011. Likewise, moderate-diversity Other neighbourhoods materialised as a new neighbourhood category in 2011. The dispersed geographies of the Chinese ethnic group (Catney, 2016a) means that we do not see Chinese majority neighbourhoods at all.

Moderately diverse Pakistani majority neighbourhoods were the most common moderately diverse category for BAME groups since 2001, and their count increased between 1991 and 2011. Moderate-diversity, rather than low-diversity, was by far, and consistently, the most common diversity type for any neighbourhood that had a majority BAME group. As with White spaces, increased diversity, rather than segregation, was the dominant trend for majority minority neighbourhoods.

While moderately diverse neighbourhoods dominated by an ethnic minority group are rare between 1991 and 2011, the steady growth of the neighbourhood type that is the main interest here is striking. High-diversity – multi-ethnic – neighbourhoods grew from just over 0.5% of the total in 1991, to over 1.5% in 2001, to over 4% in 2011. While on first consideration this may seem small, it represents the most common neighbourhood type that is not White majority. Our classification scheme’s entropy threshold and the maximum share constraints for the largest two ethnic groups necessitates that at least *five* ethnic groups must be present in these neighbourhoods. Thus, by 2011, 1,417 of England’s neighbourhoods were profoundly ethnically diverse.

**FIGURE 1:**
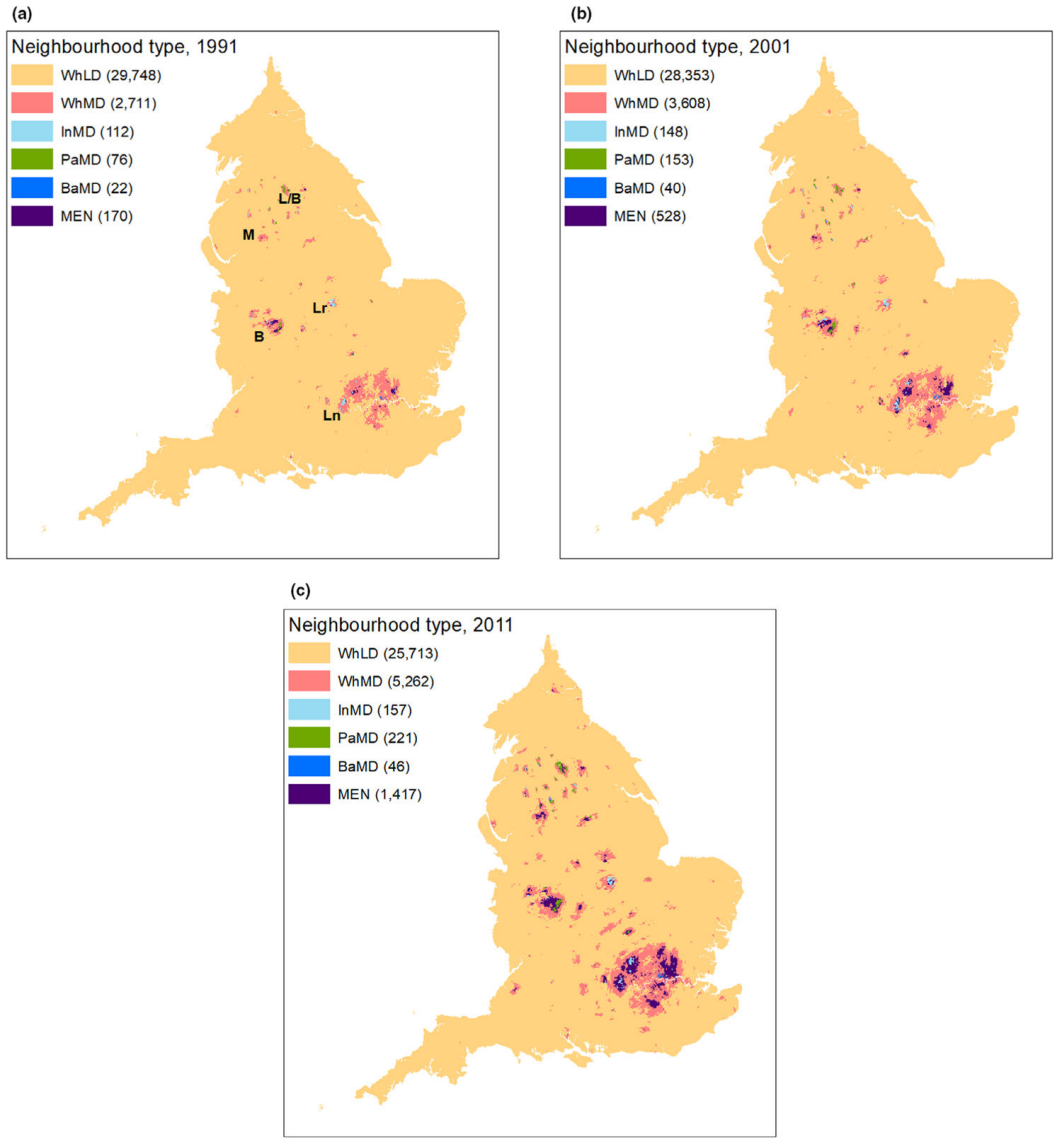
Geographical distribution of neighbourhood types: (a) 1991, (b) 2001, and (c) 2011. *Notes:* Only neighbourhood types with ten or more LSOAs in that category are shown. WhLD = Low-diversity White; WhMD = Moderate-diversity White; InMD = Moderate-diversity Indian; PaMD = Moderate-diversity Pakistani; BaMD = Moderate-diversity Bangladeshi; MEN = Multi-ethnic (high-diversity). Place names referred to in the text are labelled in approximate positions: Ln = London; B = Birmingham; Lr = Leicester; M = Manchester; L/B = Leeds and Bradford. North arrows and scale bars are not included since these maps are cartograms that distort Euclidean space *Sources*: 1991 Census, Table SAS06 (Crown Copyright); 2001 Census, Table KS006 (Crown copyright); 2011 Census, Table KS201EW (Crown Copyright)

Figure 1 shows the geography of these neighbourhoods. The maps are cartograms^3^ that, in our case, rescale the LSOAs by the square root of their original area (see Harris, 2016; Harris et al., 2017). This approach, compared to maps of Euclidean space, substantially improves the visibility of multi-ethnic neighbourhoods, particularly given their concentration in urban areas where there is a higher density of LSOAs. For the sake of clarity, only neighbourhood types with ten or more LSOAs in that category are shown.

Low diversity is the almost exclusive domain of people claiming White ethnicity in England. Yet the geography of ethnic *diversity* is also clear. In 1991, moderate-diversity White neighbourhoods were found mostly in large cities, especially London and Birmingham. By 2011, these neighbourhoods occurred in convincing numbers across much of the urbanised Midlands and north of England, as well as throughout large areas of central and greater London. South Asian low- and moderate-diversity neighbourhoods were concentrated in northern towns and cities (e.g., Bradford, Manchester), the Midlands (e.g., Leicester, Birmingham), and in east London.

The growth of multi-ethnic neighbourhoods is a key feature of England’s evolving ethnic landscape, especially in London. London was home to over 70% of England’s multi-ethnic neighbourhoods in 2011, up from just over 50% in 1991.^4^ Yet while multi-ethnic neighbourhoods were concentrated in the capital, and to a lesser degree, Birmingham, a steady growth of high-diversity neighbourhoods has occurred in other places. The number of multi-ethnic neighbourhoods grew almost threefold between 2001 and 2011, with London’s proportion remaining stable over this decade. Therefore, by 2011, multi-ethnic neighbourhoods existed in sizeable numbers outside of London, in parts of Birmingham, Greater Manchester, Bradford, Leeds, Leicester, and other metropolitan places in mid- and northern England. As we shall see, most of these transitions to high diversity came from moderately diverse White neighbourhoods. But we turn next to the question of the population composition of multi-ethnic neighbourhoods.

## 5 ∣ WHO LIVES IN MULTI-ETHNIC NEIGHBOURHOODS?

Table 2 provides insight into the demography of multi-ethnic neighbourhoods in 2011. The first two data columns of Table 2a include population counts and percentages for each ethnic group in England. The next two show the contribution of each ethnic group to the total population of multi-ethnic neighbourhoods – in other words, the number and proportion of people in each ethnic group living in these areas. The final column is the proportion of each ethnic group, of that ethnic group’s total (England) population, that live in multi-ethnic neighbourhoods. Put differently, this shows what proportion of each ethnic group are *exposed* to high diversity in their residential environment. Table 2b uses the same structure but disaggregates the White category into its constituent four ethnic groupings.

Of course, every multi-ethnic neighbourhood will tell a different story, but there is much to be learned from these summary data, and we concentrate on a few particularly interesting points. Most fundamentally, the total population living in multi-ethnic neighbourhoods in 2011 was over 2.5 million, equating to about 5% of the total population of England (Table 2a). The next column uses this total as a denominator for each ethnic group, to reveal their proportionate share in multi-ethnic neighbourhoods. In 2011, people who chose to identify with one of the ethnic groups in the “Other” category (see notes for Table 2) made up 20% of multi-ethnic neighbourhood populations. Beyond this group, multi-ethnic neighbourhoods’ largest ethnic minority membership is Indian, representing about 13% of the population of this neighbourhood type. People in the White ethnic category contributed over twice that proportion, at nearly 32%; Chinese-origin people contributed the smallest. Ethnic group share in multi-ethnic neighbourhoods thus tends to reflect group size, in rank but not in terms of their relative proportions; the largest ethnic minority group populations are considerably greater as a proportion of the population in these areas compared to their proportion of England’ s population.

The final column is arguably the most revealing in relation to the dynamics of mixing and inter-ethnic contact because it illustrates daily exposure to “lived difference” or “everyday multiculture” (Neal et al., 2013; Valentine, 2008). A considerable proportion of England’s Bangladeshi population lived in multi-ethnic neighbourhoods; indeed, nearly 32% of this group lived in mixed spaces in 2011. Remember that these areas are not low-diversity, or even moderate-diversity, Bangladeshi majority neighbourhoods – they are spaces where there is a significant *mix* of ethnic identities. This flies in the face of common perceptions of Muslim self-segregation and isolation (for further critiques see Gale, 2013; Phillips, 2015). A similarly high proportion of inter-ethnic residential mixing is demonstrated by the two Black groups (African and Caribbean).

One is also drawn to the equivalent figures for the White ethnic groups. Fewer than 2% of White people lived in multi-ethnic neighbourhoods in 2011. Drilling down further, the 2011 data unveil information on the White British, White Irish, White Gypsy or Irish Traveller, and Other White ethnic groups (Table 2b). The latter group includes high proportions of people with recent origins in ‘A8’ Accession countries, including Poland (ONS, 2013). Nearly 10% of England’s Other White population lived in multi-ethnic neighbourhoods in 2011. While only 1.25% of England’s White British population lived in multi-ethnic neighbourhoods, this translates to over 20% of the population of multi-ethnic neighbourhoods, or nearly 530,000 people. The share of England’s White British population living in multi-ethnic neighbourhoods more than doubled between 2001 and 2011 (despite an overall population decline for this group, particularly in London; Catney, 2016b).

## 6 ∣ NEIGHBOURHOOD TRANSITIONS AND (IN)STABILITY

Are multi-ethnic residential neighbourhoods in England stable or, as in the USA, transitionary between other neighbourhood types? Table 3 shows neighbourhood transitions between (a) 1991 and 2001, and (b) 2001 and 2011. Before concentrating on multi-ethnic neighbourhoods, we start with a couple of general observations. As noted, low-diversity White dominated LSOAs were by far the most common category at all three time points. The stability of low-diversity White neighbourhoods is very apparent in Table 3, with over 95% remaining in that category between 1991 and 2001. This stability weakens only slightly for transitions between 2001 and 2011, with low-diversity White neighbourhoods remaining dominant during that decade. Similarly, most neighbourhoods categorised as moderate-diversity White retained that categorisation between 1991 to 2001, and 2001 to 2011.

There are transitions, however. Unsurprisingly, the most common transition pathway is from low- to moderate-diversity White, but the second is notable: into high-diversity from moderately diverse majority White areas. From 1991 to 2001 to 2011, the count of multi-ethnic neighbourhoods grew from 170 to 528 to 1,417. Some 356 of 2001’s new multi-ethnic neighbourhoods were moderate-diversity White in 1991. The subsequent 10 years shows a similar transition pathway; 857 of the 913 emergent multi-ethnic neighbourhoods in 2011 were moderate-diversity White in 2001. Some 70% of this transition type occurred in London between 2001 and 2011, compared with 80% for the previous decade.

What were the characteristics of these formerly White spaces? Perhaps not surprisingly, neighbourhoods that transitioned from moderately diverse White majority in 2001 into high diversity in 2011 were less White than the average moderate-diversity White neighbourhood to begin with. White people constituted 55% of these transition neighbourhoods, compared to just over 65% of all 2001 moderate-diversity White neighbourhoods. For other ethnic groups, the population compositions of these transition neighbourhoods compared to all moderate-diversity White neighbourhoods ranged from no discernible difference (Chinese; 0.12% lower) to just under 3% higher (Indian).

The main transitions *away* from high diversity are relatively few and produced moderate-diversity Pakistani (17) neighbourhoods between 1991 and 2001, and moderate-diversity Pakistani (10) and Indian (9) neighbourhoods in the following decade. In stark contrast to the USA, multi-ethnic neighbourhoods in England are remarkably stable. Of the 170 multi-ethnic neighbourhoods identified in 1991, 149 (88%) had retained that status by 2001. The stability of multi-ethnic neighbourhoods increased in the subsequent 10 years. Some 504 multi-ethnic neighbourhoods, or over 95% of multi-ethnic neighbourhoods in 2001, were highly diverse in 2001 *and* 2011. Most English multi-ethnic neighbourhoods are not avenues to another type of space.

Just as London dominated the story of multi-ethnic neighbourhood formation, the capital takes the lead in neighbourhood stability. Of all neighbourhoods that retained their high-diversity state between 2001 and 2011, some 73% were in the capital. This was a significant increase on the period 1991 to 2001, where just 56% of that stability occurred in London’s multi-ethnic neighbourhoods. In other words, as London’s diversity has grown, at the neighbourhood scale, it has become *less* transitory.

## 7 ∣ THE DRIVERS OF STABLE MIXING

**FIGURE 2:**
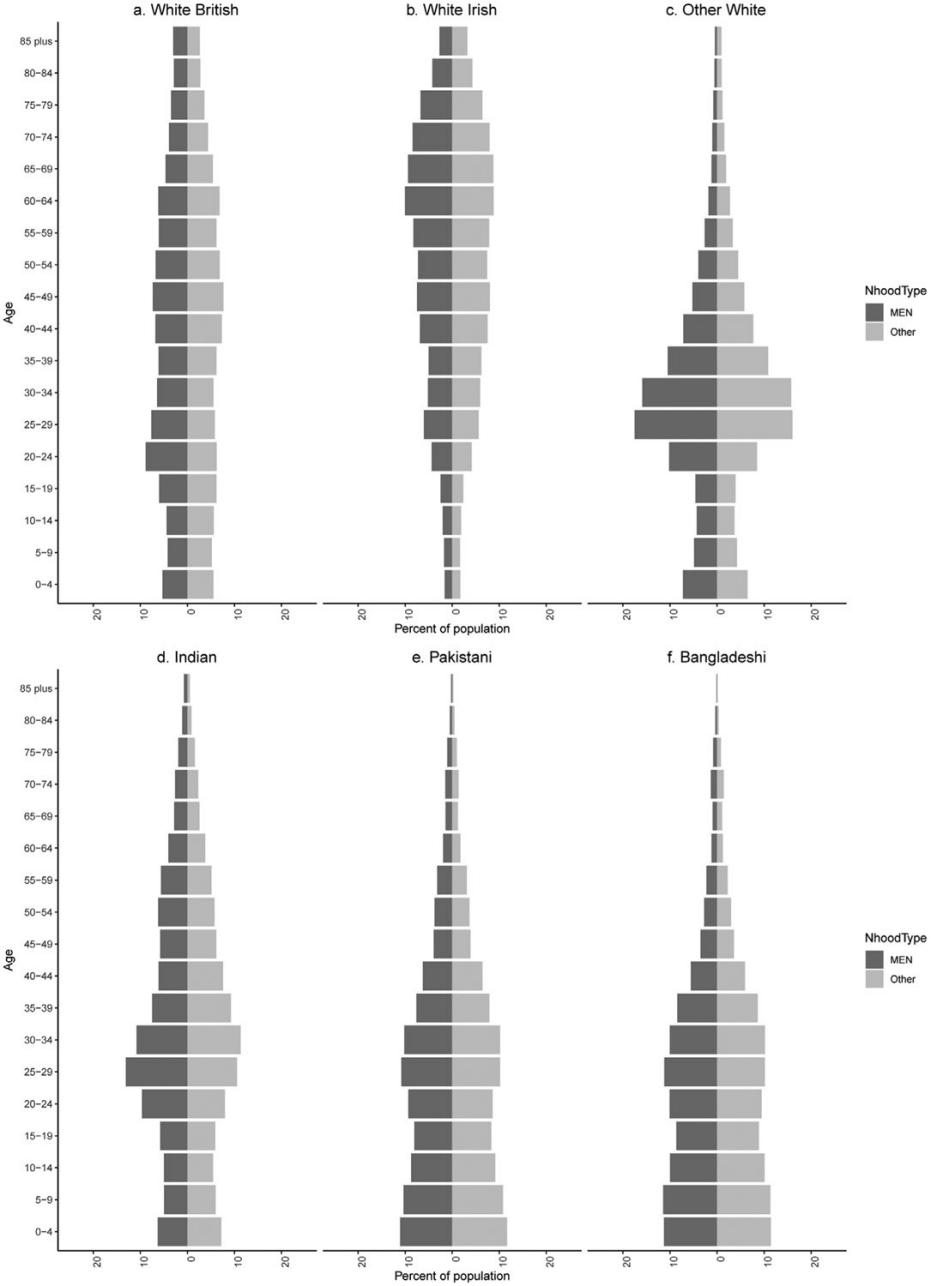

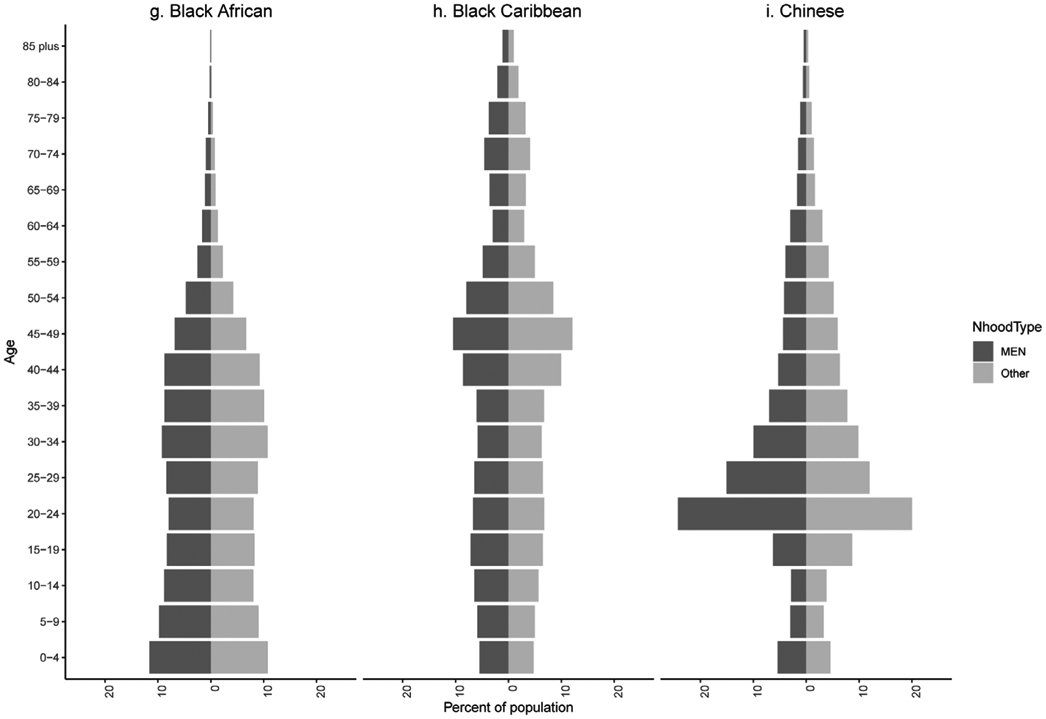
Age profiles of multi-ethnic and other neighbourhood types for selected ethnic groups in 2011: (a) White British, (b) White Irish, (c) Other White, (d) Indian, (e) Pakistani, (f) Bangladeshi, (g) Black African, (h) Black Caribbean, and (i) Chinese. *Notes*: NhoodType = neighbourhood type. MEN = multi-ethnic neighbourhood. Total populations for each ethnic group are given in Table 2 *Source*: 2011 Census, Table LC2109EWls (Crown Copyright)

To better understand the mechanisms behind the stability of English multi-ethnic neighbourhoods, we next examine the age of their residents and their housing profiles. This choice is influenced by fundamental demographic concepts. We would expect that stable neighbourhoods would be older on average and have relatively fewer renters. But what about stable *multi-ethnic* neighbourhoods? Figure 2 shows, for 2011, the age profiles of multi-ethnic neighbourhoods compared to all other neighbourhood types, by ethnic group with the exception of the heterogeneous “Other.” We also disaggregated the White ethnic category into its three largest groups: White British, White Irish, and Other White. The denominator for each side of the population pyramids is the total population in that neighbourhood type, thus indicating, for each group, what proportion of the population of multi-ethnic (and other) neighbourhoods are, for example, young or old adults.

The age profiles of multi-ethnic neighbourhoods are, on average, slightly younger than other neighbourhood types, with a higher proportion of their population in their 20s and 30s. Students, however, are not dominating this story. For example, while a large share of the Chinese population living in multi-ethnic neighbourhoods are aged 20–24, this equates to a small fraction of the *overall* Chinese population in this age range (9,121 people in multi-ethnic neighbourhoods compared to 68,507 in other neighbourhood types). Also of note are the relatively large shares of people across *several* younger age groups, from young children to those in their early 40s, living in multi-ethnic neighbourhoods.

Disaggregation of the White category sheds light on whether White youthfulness in multi-ethnic neighbourhoods is attributable to the younger Other White population, with origins in recent immigration. A considerable proportion of this group can indeed be found in multi-ethnic neighbourhoods (as for other neighbourhoods), yet multi-ethnic neighbourhoods are also home to a relatively large share of young adult White Britons. Thus while ethnic minority groups typically have younger age structures than the White British (and White Irish) populations (Simpson & Jivraj, 2015), the White British population of multi-ethnic neighbourhoods is, as with ethnic minority groups, youthful. The results do not suggest that older Whites are ageing out of these places; instead, they are indicative of population *replacement*, likely through dual processes of natural change and in-migration. Rather than the growth of a single group, or groups, representing a pathway towards new forms of non-White segregation or gentrification by in-migrating White people – as in the USA – in the English case, the population profiles of multi-ethnic neighbourhoods suggest stable *mixing* among White and minority groups.

Our consideration of housing relates to levels of home ownership in multi-ethnic neighbourhoods, a tenure traditionally associated with less mobility and greater commitment to neighbourhood than (particularly private) renting. Table 4 shows the proportion of the 2011 population in each tenure for multi-ethnic neighbourhoods and all other neighbourhood types, for all of England and, separately, for neighbourhoods in London and the rest of England.

Home-ownership rates were particularly low for multi-ethnic neighbourhoods in 2011, compared to other neighbourhood types. Just 42% of the population in multi-ethnic neighbourhoods in England owned their property (either outright, or with a mortgage), compared to nearly 67% of residents of other neighbourhood types. In contrast, both social and private renting rates were very high in multi-ethnic neighbourhoods. This is a persistent observation from 1991 (not shown given space constraints).

The relatively low rates of owner-occupation observed for multi-ethnic neighbourhoods might be explained by the fact that, given discrimination, inequalities, and differential accommodation preferences, BAME populations are disproportionately represented in private or social rented housing, an issue particularly common in London, which has some of the highest costs of housing globally (GLA, 2018). In the UK, private renting is often a precarious tenure, subject to poor quality housing and landlord whims on tenancy, rent prices, and maintenance. Additional explanations might include the urban dominance of both renting (particularly social) *and* multi-ethnicity, and the younger age profiles of these neighbourhoods (Figure 2). London’s rates of social and private renting are particularly high; while outside of London rates of private renting are over 10% greater in multi-ethnic than other neighbourhoods, the difference between London’s multi-ethnic and other neighbourhoods is not so marked (30% compared to 26%, respectively). The results suggest that the stability of multi-ethnic neighbourhoods is not a function of “rootedness” to place brought about by home-ownership, or middle-class gentrification. Rather, two alternative mechanisms might be at play. First, stability of neighbourhood type might reflect loyalty to place (see Frost and Catney (2020) for an example outside of London), or, less positively, a “stickiness” within multi-ethnic neighbourhoods as a function of barriers in the housing market. Second, and alternatively, there may be high levels of migration into and out of multi-ethnic neighbourhoods – a stability, therefore, of *area* type, rather than of *population*. This resonates with Skifter Andersen’s (2010) study of Danish multi-ethnic neighbourhoods, in which immigrants initially settled before moving on to other neighbourhoods.

## 8 ∣ DISCUSSION AND CONCLUSIONS

This paper had two main goals: to characterise highly ethnically diverse neighbourhoods in England and to analyse how they came to be and the extent of their stability. While White majority spaces prevail, low-diversity White-dominated neighbourhoods are being gradually succeeded by more mixed spaces, with most becoming moderately diverse White neighbourhoods. In turn, a growing proportion of these neighbourhoods turned over into the most diverse and ethnically mixed type – multi-ethnic neighbourhoods. Employing a classification of these neighbourhoods whereby at least five ethnic groups need to be present and no one ethnic group can dominate, we have been able to show the evolution of highly diverse spaces over time.

The most important result is that multi-ethnic neighbourhoods in England are stable, a finding in stark contrast to the experience of equivalent high-diversity spaces in the USA (Wright et al., 2020a). While in the USA less than 50% of neighbourhoods retained their high-diversity status for more than a decade (2000–2010), in England fully 95% of high-diversity neighbourhoods retained that status between 2001 and 2011. This is even more remarkable when we consider how the spatial units that define neighbourhoods in this study have considerably smaller mean populations (1,600 people) than the US Census tracts (~4,000) that denote neighbourhoods in Wright et al. (2020a). An intuitive hypothesis is that smaller neighbourhoods would have less stability, but we find the opposite.

The picture we paint of stably mixed, multi-ethnic neighbourhoods leads us to speculate that these locales may be acting as magnets for diversity, whereby population churn and replacement might contribute to the stability of neighbourhood type (rather than of people). Are we observing layers of ethnic diversity as populations move in and out of these neighbourhoods, according to their housing, employment, and education needs? Future research making use of longitudinal data to explore length of stay in these places would shed further light on these processes. The evidence presented here suggests a different evolution – and retention – of high diversity in England compared to the USA, and therefore different processes at work in each context.

Our story also challenges dominant UK policy-political narratives of increasing diversity as a pathway to increasing polarisation (Casey, 2016). Increased diversity, rather than segregation, is the direction of travel for almost all neighbourhood types. Contrary to popular debate, “South Asian” majority residential spaces – Indian, Pakistani, and Bangladeshi areas – are becoming moderately diverse, not low-diversity. Our observed transitions into moderate- and ultimately high-diversity are suggestive that areas classified as “segregated” at one point in time could well be in transition to greater ethnic diversity – *and* might well retain that status. Likewise, based on the observed trajectories, there is no evidence of an emergence of low-diversity non-White spaces in the future. Indeed, some moderate-diversity White-dominated neighbourhoods were transitioning into other forms of diversity (most commonly into high-diversity). It therefore might be reasonable to expect that these shifts will be associated with new forms of diversity.

The youthful age profiles of multi-ethnic neighbourhoods across *all* – White British and ethnic minority – groups, despite differing population profiles at the national level (Simpson & Jivraj, 2015), suggest a stable mixing that is likely to continue. We therefore concur with Simpson’s prediction that “the future of Britain is a greater variety of diverse areas” (2015, p. 49; see also Lomax et al., 2020). While there is no doubt that global cities are unequal and becoming more so, that inequality is racialised, and that segregation persists in certain locales, our findings do not support the “dual city” label attached to some areas (see MacFarlane & Mitchell, 2019). Indeed, the rise and stability of multi-ethnic neighbourhoods in London signals that such binaries ignore the complexity of neighbourhood experiences in large and ethnically diverse cities (e.g., Crul, 2016).

Multi-ethnic spaces are worthy of attention not only because they challenge dominant stereotypes of increasing ethnic diversity as increasing ethnic segregation, but also because they are examples of multiculturalism and spatial integration in practice. They offer greater opportunities for “everyday” inter-ethnic contact (Neal et al., 2013), which may serve to drive down prejudice and intolerance (Allport, 1954), and provide support and a sense of belonging for new and established groups (Peach, 1996; Skifter Andersen, 2010). (Diverse) cities might thus be viewed as “reservoirs of hope” (Thrift, 2005, p. 147). Between 2001 and 2011, the population of mixed ethnic groups nearly doubled, to over 1.2 million (Catney, 2016b). As spaces where people of different ethnic groups come together and potentially even form romantic unions, multi-ethnic neighbourhoods might be understood as “places of possibility” for future inter-ethnic household mixing and the emergence of multi-ethnic identities (Houston et al., 2005).

But while the assets of diversity might be realised in these spaces, our optimism is tempered by some caution. Valentine urges us to be wary of over-romanticising the impact of everyday encounter, where residents are “living with difference” (2008, p. 323), in nurturing inter-ethnic (or, more generally, inter-group) tolerance. Amin (2002) and Walton and Hardebeck (2016) suggest the need for more proactive measures to encourage meaningful inter-ethnic contact rather than simply sharing residential space. Robinson (2005) warns that residential integration does not necessarily lead to social interaction, and that certain conditions are required in the nature of the contact before attitudinal changes take place (see also Bynner, 2019). Karner and Parker (2011) reveal the complexities of interactions, conflicts, and (positive) experiences in a diverse neighbourhood in Birmingham.

Additionally, the uncertainties posed by the impact of Brexit loom large. The implications of leaving the European Union (EU) on future immigration/emigration scenarios are difficult to predict, and every possible scenario will have at least some impact on the UK’s diverse future (Lomax et al., 2020). Reduced population growth for some ethnic groups would potentially have consequences for the future stability of multi-ethnic neighbourhoods. Added to this, the decision to leave the EU is associated with increased racial violence (Burnett, 2017), which could have major implications for feelings of being welcome in diverse spaces, as well as their stability.

## Figures and Tables

**TABLE 1:** Neighbourhood types in England, 1991, 2001, and 2011

Neighbourhood type	1991 *n*	2001 *n*	2011 *N*	1991 %	2001 %	2011 %
Low-diversity White	29,748	28,353	25,713	90.57	86.33	78.29
Moderate-diversity White	2,711	3,608	5,262	8.25	10.99	16.02
Low-diversity Indian	5	7	8	0.02	0.02	0.02
Moderate-diversity Indian	112	148	157	0.34	0.45	0.48
Low-diversity Pakistani	0	2	6	0.00	0.01	0.02
Moderate-diversity Pakistani	76	153	221	0.23	0.47	0.67
Low-diversity Bangladeshi	0	2	2	0.00	0.01	0.01
Moderate-diversity Bangladeshi	22	40	46	0.07	0.12	0.14
Moderate-diversity Black African	0	3	3	0.00	0.01	0.01
Moderate-diversity Other	0	0	9	0.00	0.00	0.03
Multi-ethnic	170	528	1,417	0.52	1.61	4.31
Total	32,844	32,844	32,844	100	100	100

*Note*: *n* = number of neighbourhoods; % = percentage of total for England

*Sources*: 1991 Census, Table SAS06 (Crown Copyright); 2001 Census, Table KS006 (Crown copyright); 2011 Census, Table KS201EW (Crown Copyright). The diversity classification is devised by the authors using the official statistics

**TABLE 2:** Multi-ethnic neighbourhood populations in England by ethnic group, in 2011

(a) 2011, all ethnic groups					
Ethnic group	England pop.	% England pop.	MEN pop.	% MEN pop.	% pop. living in MEN
White	45,281,142	85.42	809,912	31.67	1.79
Indian	1,395,702	2.63	334,542	13.08	23.97
Pakistani	1,112,282	2.10	276,188	10.80	24.83
Bangladeshi	436,514	0.82	138,578	5.42	31.75
Chinese	379,503	0.72	37,608	1.47	9.91
Black African	977,741	1.84	268,099	10.48	27.42
Black Caribbean	591,016	1.11	175,997	6.88	29.78
Other	2,838,556	5.35	516,342	20.19	18.19
Total	53,012,456	100	2,557,266	100	n/a
**(b) 2011, White ethnic groups**					
White groups	England pop.	% England pop.	MEN pop.	% MEN pop.	% pop. living in MEN
White British	42,279,236	79.75	527,427	20.62	1.25
White Irish	517,001	0.98	38,414	1.50	7.43
White Gypsy or Irish Traveller	54,895	0.10	3,189	0.12	5.81
Other White	2,430,010	4.58	240,882	9.42	9.91
Total	45,281,142	85.42	809,912	31.67	n/a

*Notes*: MEN = multi-ethnic neighbourhood. Other comprises, in 2011: Mixed White and Black Caribbean, Mixed White and Black African, Mixed White and Asian, Other Mixed, Other Asian, Other Black, Arab, Any other ethnic group. n/a: not applicable. Due to rounding, percentages may not sum to 100

*Source*: 2011 Census, Table KS201EW (Crown Copyright)

**TABLE 3:** Neighbourhood transition matrices, (a) 1991–2001 and (b) 2001–2011

(a)	2001											
	Diversity category	LD White	MD White	LD Indian	MD Indian	LD Pakistani	MD Pakistani	LD Bangladeshi	MD Bangladeshi	MD Black African	MEN	Total
1991	LD White	28,307	1,439	0	0	0	0	0	0	0	2	29,748
	MD White	46	2,161	0	58	0	63	0	24	3	356	2,711
	LD Indian	0	0	5	0	0	0	0	0	0	0	5
	MD Indian	0	2	2	89	0	2	0	0	0	17	112
	MD Pakistani	0	1	0	0	2	71	0	0	0	2	76
	MD Bangladeshi	0	2	0	0	0	0	2	16	0	2	22
	MEN	0	3	0	1	0	17	0	0	0	149	170
	Total	28,353	3,608	7	148	2	153	2	40	3	528	32.844

(b)	2011												
	Diversity category	LD White	MD White	LD Indian	MD Indian	LD Pakistani	MD Pakistani	LD Bangladeshi	MD Bangladeshi	MD Black African	MD Other	MEN	Total
2001	LD White	25,699	2,648	0	1	0	3	0	1	0	0	1	28,353
	MD White	14	2,604	0	35	0	74	0	13	3	8	857	3,608
	LD Indian	0	0	7	0	0	0	0	0	0	0	0	7
	MD Indian	0	0	1	111	0	1	0	0	0	0	35	148
	LD Pakistani	0	0	0	0	2	0	0	0	0	0	0	2
	MD Pakistani	0	1	0	1	4	133	0	0	0	0	14	153
	LD Bangladeshi	0	0	0	0	0	0	1	1	0	0	0	2
	MD Bangladeshi	0	6	0	0	0	0	1	30	0	0	3	40
	MD Black African	0	0	0	0	0	0	0	0	0	0	3	3
	MEN	0	3	0	9	0	10	0	1	0	1	504	528
	Total	25,713	5,262	8	157	6	221	2	46	3	9	1,417	32,844

*Note*: LD = low-diversity; MD = moderate-diversity; MEN = multi-ethnic neighbourhood

*Sources*: 1991 Census, Table SAS06 (Crown Copyright); 2001 Census, Table KS006 (Crown copyright); 2011 Census, Table KS201EW (Crown Copyright)

**TABLE 4:** Proportion of the population in multi-ethnic and all other neighbourhoods by housing tenure, 2011

Region	Tenure	Owner-occupation		Social renting		Private renting	
	Neighbourhood type	MEN	Other	MEN	Other	MEN	Other
England		42.15	66.71	28.91	15.83	28.93	17.46
London		40.55	52.58	29.85	21.12	29.60	26.30
Rest of England		46.14	68.76	26.59	15.06	27.28	16.18

*Notes*: MEN = multi-ethnic neighbourhood. Data are reported for all usual residents in households (all other data are for all usual residents)

*Source*: 2011 Census, Table DC4203EW (Crown Copyright)

## Data Availability

The data that support the findings of this study are openly available in Casweb (UK Data Service) at http://casweb.ukdataservice.ac.uk/ (1991 and 2001 data) and Nomis: Official Labour Market Statistics (Office for National Statistics) at https://www.nomisweb.co.uk/census/2011 (2011 data). The 1991 and 2001 ethnic group population estimates for 2011 Lower Layer Super Output Areas are freely available at https://www.qub.ac.uk/research-centres/GIS/Research/PopChange/Data/

## Footnotes

1 See https://journals.sagepub.com/etna/symposium

2 The base units for 1991 were Enumeration Districts (EDs; mean population = 420), the smallest available output zones for that Census year. Census data for 1991 were linked to 2011 LSOAs using areal weighting (overlay), with postcode data used to more precisely allocate counts from EDs to overlapping (parts of) LSOAs. The result is a set of counts for 1991, for 2011 LSOAs. Note that, partly because LSOAs were designed with respect to 2011 populations, some LSOAs had small populations in 1991 since the overlapping parts of EDs had very small populations (likely representing areas of subsequent population growth). The 2001 LSOA counts were assigned to 2011 LSOAs using the lookup table provided by the Office for National Statistics (see https://data.gov.uk/dataset/afc2ed54-f1c5-44f3-b8bb-6454eb0153d0/lower-layer-super-output-area-2001-to-lower-layer-super-output-area-2011-to-local-authority-district-2011-lookup-in-england-and-wales). Computing entropy requires logs of percentages. Since some groups are absent in some LSOAs and the natural logarithm of zero is undefined, a constant must be added to the counts. For 2001 and 2011, a constant of 0.1 was added to all ethnic group counts prior to computing percentages. For 1991, a constant of 0.1 was added to all counts except for those LSOAs estimated to have ten or fewer people. In these cases, the constant was 0.0000001. The results are stable to changes in the constant. The data are freely available at https://www.qub.ac.uk/research-centres/GIS/Research/PopChange/Data/

3 Cartograms are described in Dorling (1996). A cartogram shapefile was created for this study using open software at http://scapetoad.choros.place/

4 Our definition of London is all LSOAs located within the London region.
